# A survey of Alabama eye care providers in 2010–2011

**DOI:** 10.1186/1471-2415-14-44

**Published:** 2014-04-03

**Authors:** Paul A MacLennan, Gerald McGwin, Karen Searcey, Cynthia Owsley

**Affiliations:** 1 Department of Surgery, School of Medicine, University of Alabama at Birmingham, 115 Kracke Building, 1530 3rd Ave South, Birmingham, AL 35294-0016, USA; 2 Department of Ophthalmology, School of Medicine, University of Alabama at Birmingham, Suite 609, 700 South 18th Street, 35294-0009 Birmingham, AL, USA

**Keywords:** Epidemiology, Survey, Vision care, Diabetic retinopathy

## Abstract

**Background:**

State level information regarding eye care resources can provide policy makers with valuable information about availability of eye care services. The current study surveyed ophthalmologists, optometrists and vision rehabilitation providers practicing in Alabama.

**Methods:**

Three mutually exclusive provider groups were identified, i.e., all ophthalmologists, optometrists, and vision rehabilitation providers working in Alabama in 2010. Eligible providers were contacted in 2010 and 2011 and information was requested regarding provider demographics and training, practice type and service characteristics, and patient characteristics. Descriptive statistics (e.g., means, proportions) were used to characterize provider groups by their demographic and training characteristics, practice characteristics, services provided and patients or clients served. In addition, county level figures demonstrate the numbers and per capita ophthalmologists and optometrists.

**Results:**

Ophthalmologists were located in 24 of Alabama’s 67 counties, optometrists in 56, and 10 counties had neither an ophthalmologist nor an optometrist. Overall, 1,033 vision care professionals were identified as eligible to participate in the survey: 217 ophthalmologists, 638 optometrists, and 178 visual rehabilitation providers. Of those, 111 (51.2%) ophthalmologists, 246 (38.6%) optometrists, and 81 (45.5%) rehabilitation providers participated. Most participating ophthalmologists, optometrists, and vision rehabilitation providers identified themselves as non-Hispanic White. Ophthalmologists and optometrists estimated that 27% and 22%, respectively, of their patients had diabetes but that the proportion that adhered to eye care guidelines was 61% among ophthalmology patients and 53% among optometry patients.

**Conclusions:**

A large number of Alabama communities are isolated from eye care services. Increased future demand for eye care is anticipated nationally given the aging of the population and decreasing numbers of providers; however, Alabama also has a high and growing prevalence of diabetes which will result in greater numbers at risk for diabetic retinopathy, glaucoma, and cataracts.

## Background

Vision health is an important public health concern that affects people of all ages in the United States with annual costs from medical treatment and disability of approximately $50 billion (US) [1]. For eye diseases such as cataract, glaucoma, age-related macular degeneration, and diabetic retinopathy, inadequate access to vision care, i.e., assessment, diagnosis, treatment and rehabilitation, results in delayed diagnosis, and unnecessary increases in burden of disease, disability and costs [1-3]. Barriers to vision care are related to uninformed attitudes about the importance of routine eye care, i.e., thinking there is no need, costs, and accessibility [4]. Information regarding the distribution and characteristics of eye care resources can be used to identify areas in need and inform policy makers in their decisions regarding eye care services.

Compared to many other states, Alabama has a disproportionate increase in the prevalence of many risk factors associated with eye diseases. For example, Alabama’s population includes a large proportion (approximately 26%) of African Americans, and previous research indicates that rates of vision impairment and eye disease among African Americans are two times higher than those of whites, especially uncorrected refractive error, cataract, and diabetic retinopathy [5-7]. Glaucoma is 3–4 times more common in African Americans as compared to whites of non-Hispanic origin [8,9]. In addition, the prevalence of diabetes among those 16 and older in Alabama is very high (approximately 13%) and diabetic retinopathy is the leading cause of blindness among US working age adults and those with diabetes are also at increased risk for glaucoma and cataracts [9-13]. Furthermore, adults diagnosed with one of four major eye diseases and without eye care insurance less frequently followed recommended guidelines for visiting an eye-care provider; in 2011, 16% of Alabama’s population was without health insurance [14,15].

The objective of the current survey of Alabama eye care providers was to obtain information about the characteristics of providers, their practices and patients.

## Methods

The Institutional Review Board of the University of Alabama at Birmingham reviewed and approved the study protocol.

### Study population

The study population consisted of three provider groups: (1) Ophthalmologists, defined as physicians (MD or DO) who have a medical license in Alabama per the Alabama State Board of Medical Examiners, have completed residency training in ophthalmology, and practice at least part time in Alabama; (2) Optometrists, defined as those who have a Doctor of Optometry degree, are licensed by the Alabama Board of Optometry to practice optometry in Alabama, and practice at least part time in Alabama; and (3) Vision rehabilitation providers, defined as those who provide vision rehabilitation services and practice at least part time in Alabama. Ophthalmologists and optometrists who provide vision rehabilitation services were not included in the vision rehabilitation provider category.

Eligible participants were identified from August 2010 through October 2010 using information obtained from professional associations, licensing boards, and internet searches. Attempts were made to contact all potential participants via telephone to verify that providers still worked in Alabama and that their contact information was correct. When incorrect, contact information was updated; however, participants who met the exclusion criteria, e.g., retired and no longer practicing, deceased, or relocated outside of Alabama, were deemed ineligible.

### Survey content

Participants provided their demographic information (i.e., race/ethnicity, age and gender) and details regarding their practice and patients. Requested practice characteristics included type of practice (i.e., group practice with another ophthalmologist or optometrist, and whether their practice was university based, a Department of Veterans Affairs facility, rehabilitation or general hospital, outpatient rehabilitation clinic, independent service for the visually impaired, State agency, and optical retail). In addition, information was requested about other settings (i.e., day programs in public or private schools, residential schools, in-patient psychiatric or general hospital, nursing homes, and state/federal/local correctional facilities) where participants provided services. With respect to training, ophthalmologists provided information regarding the year of residency completion, whether residency was followed by a fellowship, and if yes, the field of training; from optometrists, the year of receiving optometry degree, whether residency was completed, and if yes, the field of specialty training; and from rehabilitation providers, the year of receiving highest degree and vision rehabilitation specialty.

Participating ophthalmologists and optometrists were asked whether they provided comprehensive eye care for adults, comprehensive eye care for infants and children, contact lens fitting and dispensing. Ophthalmologists were asked whether they provided cataract surgery, refractive surgery, retinal-vitreal surgery, glaucoma surgery, corneal surgery, oculo-plastic surgery, visual rehabilitation services, and neuro-ophthalmological services. Optometrists were asked whether they provided vision therapy and/or low vision rehabilitation services. Rehabilitation providers were asked whether they provided in-home services, training services (i.e., the use of assistive devices, orientation and mobility, eccentric viewing or preferred retinal loci, scanning strategy, strategies to perform everyday visual tasks, and the use of computers and software), psychological or counseling, support groups, social work, driving rehabilitation, home-based visits for education or training, and vocational rehabilitation or career counseling.

Participants provided estimates for the proportions of their patients by age group (<5, 5–19, 20–59, 60–79, and 80+), race (white, non-Hispanic, African American, Hispanic, Asian, Native American, and other), gender, and insurance type (e.g., Medicare, Medicaid, private insurance). Similarly, participants were asked to provide estimates of the prevalences of diabetes and low-vision among their patients; in addition, ophthalmologists and optometrists estimated the proportion of diabetic patients that adhere to eye care guidelines. Participating rehabilitation providers estimated the proportion of their patients with the following specific difficulties or problems: reading, writing, financial management, other detailed near tasks, independent living, mobility, driving, identification of objects/people/events from a distance, self care/domestic activity, and emotional or psychological adjustment.

### Survey conduct

Eligible participants (N = 1,033) were contacted over a ten-month period from November 2010 through August 2011 An initial mail contacted them of the study goals and requested their participation. Included in the mail contact was a survey specific to their provider type (Ophthalmologists, Optometrists, and Vision rehabilitation providers) and a pre-paid return envelope. Additional steps were taken to encourage participation among non-responders; these included: telephone calls to practices to remind the provider about the opportunity to participate, faxes and emails by study personnel to the provider, attendance at several professional seminars and conferences where surveys were made available, two announcements of the survey in the Alabama Optometric Association monthly newsletter, a mass email to members of the Alabama Optometric Association, and the option of completing the survey online.

### Statistical analysis

Descriptive statistics (e.g., means, proportions) were calculated based on the providers who responded to each specific question. For example, if there were 100 survey participants but only 90 provided gender information, the denominator used to calculate the proportions of men and women would be 90. For patient characteristics (e.g., demographics, prevalence of diabetes and low-vision), descriptive statistics were weighted by the number of patients or clients estimated to be personally seen by participants.

## Results

**Figure 1:**
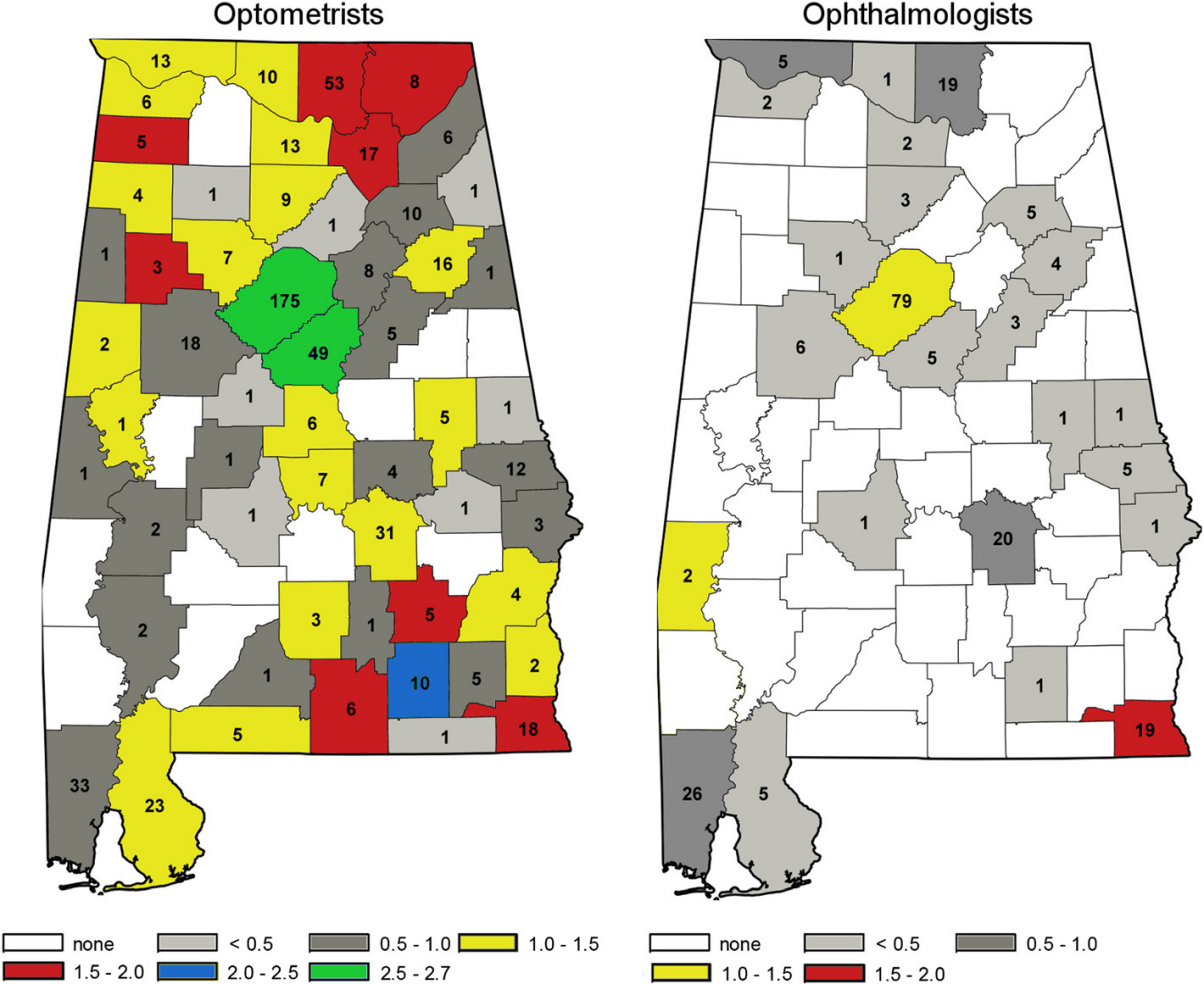
Alabama County of practice location, numbers of and per 10,000 population, optometrists and ophthalmologists.

Of the 1,033 vision care professionals that were eligible to participate in the survey (217 ophthalmologists, 638 optometrists, and 178 rehabilitation providers), 111 (51.2%) ophthalmologists, 246 (38.6%) optometrists, and 81 (45.5%) rehabilitation providers did so. Figure 1 shows the numbers of, and per capita, optometrists and ophthalmologist by county of location. The maps show that the majority of eligible providers were located in urban counties, e.g., Jefferson (175 optometrists, 79 ophthalmologists), Madison (53 optometrists, 19 ophthalmologists), Mobile (33 optometrists, 26 ophthalmologists), Shelby (49 optometrists, 5 ophthalmologists), and Montgomery (31 optometrists, 20 ophthalmologists). Of Alabama’s 67 counties, 56 had at least one optometrist, 24 at least one ophthalmologist, and ten counties had neither an ophthalmologist nor optometrist. In general, counties of the state with no or few providers were clustered in west central Alabama, known as the Black Belt region, where many counties have large African American populations.

The majority of participating ophthalmologists, optometrists, and vision rehabilitation providers identified themselves as non-Hispanic White (Table 1). Few minorities were represented in any group, though 16 (6.6%) of 241 optometrists and 11 (13.6%) of 81 vision rehabilitation providers indicated they were African American. Ophthalmologists were on average, approximately seven years older than optometrists. Fewer ophthalmologists were women (11.2%) compared to 44.3% of optometrists and 80.3% of vision rehabilitation providers.

**Table 1:** Demographic and practice characteristics of survey participants by provider group

	**Ophthalmologists**	**Optometrists**	**Rehabilitation**
Participants (N)	111	246	81
Race/ethnicity group (%)			
White, non-Hispanic	103 (94.5)	216 (89.6)	68 (84.0)
African-American	1 (0.9)	16 (6.6)	11 (13.6)
Hispanic	1 (0.9)	4 (1.7)	1 (1.2)
Asian	2 (1.8)	2 (0.8)	0 (0.0)
Native American	1 (0.9)	2 (0.8)	1 (1.2)
Other	1 (0.9)	1 (0.4)	0 (0.0)
Average age (SD)	53.0 (11.8)	45.7 (12.0)	47.8 (11.1)
Gender (%)			
Male	95 (88.8)	136 (55.7)	16 (19.8)
Female	12 (11.2)	108 (44.3)	65 (80.2)
**Practice types**			
Private practice with at least one Ophthalmologist	91 (82.0)	25 (10.2)	0 (0.0)
Private practice with at least one Optometrist	27 (24.3)	150 (61.0)	0 (0.0)
Practice based in a university	13 (11.7)	21 (8.5)	4 (4.9)
Department of Veterans Affairs clinic or medical center	3 (2.7)	10 (4.1)	11 (13.6)
Rehabilitation hospital	0 (0.0)	1 (0.4)	1 (1.2)
General hospital	4 (3.6)	3 (1.2)	0 (0.0)
Outpatient rehabilitation center	0 (0.0)	1 (0.4)	1 (1.2)
Independent service for the visually impaired	0 (0.0)	1(0.4)	11 (13.6)
State agency	0 (0.0)	2 (0.8)	44 (54.3)
Optical retail store	8 (7.2)	46 (18.7)	0 (0.0)
Other	4 (3.6)	18 (7.3)	21 (25.9)
**Other settings where services provided**			
Public or private schools (day programs)	0 (----)	12 (4.9)	21 (25.9)
Residential schools (e.g., Alabama Institute for the Deaf & Blind)	1 (0.9)	4 (1.6)	18 (22.2)
General hospitals	20 (18.2)	9 (3.7)	0 (----)
In-patient psychiatric hospitals	2 (1.8)	4 (1.6)	0 (----)
Nursing homes	3 (2.7)	28 (11.4)	8 (9.9)
State or Federal prisons or Local jails	0 (----)	7 (2.9)	3 (3.7)
Other	1 (0.9)	25 (10.2)	17 (21.0)

Most ophthalmologists (82.0%) worked in a private practice with at least one other ophthalmologist and 24.3% reported working in a practice with at least one optometrist, whereas 61.0% of optometrists worked in private practice with at least one other optometrist, and 10.2% worked in practice with at least one ophthalmologist (Table 1). Most (54.3%) vision rehabilitation providers worked for a state agency. Other settings that ophthalmologists provided services included general hospitals (18.2%); optometrists provided services at day programs at public or private schools (4.9%), residential schools (1.6%), general hospitals (3.7%), in-patient psychiatric hospitals (1.6%), nursing homes (11.4%), and federal/state/local correctional facilities (2.9%). Rehabilitation providers also reported performing services at day programs at public or private schools (25.9%), residential schools (22.2%), nursing homes (9.9%), and federal/state/local correctional facilities (3.7%).

Among ophthalmologists, 25.2% completed their residency training in or after 2000 and 35.2% before 1980 (Table 2). Almost 50% of ophthalmologists had completed a fellowship, and among those, the most common areas of specialty were retina (20.4%), cornea (18.5%), glaucoma (16.7%), pediatric ophthalmology (13.0) and oculoplastics (13%). Among optometrists, 31.7% received their optometry degree in or after 2000 and 16.7% before 1980. Of those who reported completing a residency (21.3%), the most frequently areas of specialty training were family practice (26.9%), geriatric optometry (26.9%), low vision rehabilitation (23.1%) and primary eye care (21.1%). Among rehabilitation providers, 33.3% received their highest degree in 2000 or after and 24.7% before 1980. Rehabilitation providers’ specialties included educator (30.7%), vision rehabilitation therapist (20.0%), rehabilitation counselor (16.0%), and vocational rehabilitation counselor (9.3%). Very few participants identified themselves as low vision therapists (5.3%); however, many participants specified another specialty (30.7%) outside of the ones offered in the survey, e.g., case management.

**Table 2:** Training characteristics of ophthalmologists and optometrists

**Ophthalmologists (N = 111)**	**N (%)**
**Year completed – residency training**	
2000 and after	28 (25.2)
1990 – 1999	22 (19.8)
1980 – 1989	22 (19.8)
Before 1980	39 (35.2)
**Fellowship post residency?**	
Yes (%)	53 (49.5)
**If yes, in what field (%)**	
Retina	11 (20.4)
Glaucoma	9 (16.7)
Cornea	10 (18.5)
Pediatric Ophthalmology & Strabismus	7 (13.0)
Neuro-ophthalmology	4 (7.4)
Oculoplastics	7 (13.0)
Visual rehabilitation	0 (--)
Ophthalmic pathology	1 (1.9)
Ocular inflammatory disease	1 (1.9)
Other	7 (13.0)
**Optometrists (N = 246)**	
**Year O.D. received**	
2000 and after	78 (31.7)
1990 – 1999	67 (27.2)
1980 – 1989	60 (24.4)
Before 1980	41 (16.7)
**Following O.D., residency in specialty?**	
Yes (%)	52 (21.3)
**If yes, in what specialty?**	
Community health optometry	0 (--)
Cornea and contact lenses	5 (9.6)
Family practice optometry	14 (26.9)
Geriatric Optometry	14 (26.9
Low vision rehabilitation	12 (23.1)
Pediatric optometry	4 (7.7)
Primary eye care	11 (21.1)
Refractive and ocular surgery	1 (1.9)
Vision therapy	2 (3.8)
Other	16 (30.8)
**Rehabilitation providers (N = 81)**	
**Year degree received**	
2000 and after	27 (33.3)
1990 – 1999	21 (25.9)
1980 – 1989	13 (16.1)
Before 1980	20 (24.7)
**Specialty**	
Occupational therapist	7 (9.3)
Occupational therapist assistant	0 (--)
Vision rehabilitation therapist	15 (20.0)
Certified low vision therapist	4 (5.3)
Social worker	2 (2.7)
Rehabilitation counselor	12 (16.0)
Vocational rehabilitation counselor	7 (9.3)
Psychologist	2 (2.7)
Educator	23 (30.7)

Table 3 describes the services provided by ophthalmologists and optometrists. Among participating ophthalmologists, 78.2% offered comprehensive eye care for adults, and children (52.7%), as well as dispensing and fitting of contact lenses (41.8%). Approximately 80% performed any surgery, 61% performed cataract surgery but fewer performed other types of surgeries: refractive (20.0%), retinal (13.5%), glaucoma (31.8%), corneal (18.2%) and oculoplastic (33.6%). Few ophthalmologists (2.7%) provided visual rehabilitation services and 13.6% provided neuro-ophthalmological services. Among optometrists, 95.1% provided comprehensive eye care for adults, and children (81.3%), as well as fitting and dispensing contact lenses (86.2%). Optometrists reported that 12.6% provided vision therapy and 15.0% provided low vision rehabilitation services.

**Table 3:** Services provided by ophthalmologists and optometrists

**Ophthalmologists (N = 111)**	**N (%)**
Comprehensive eye care for adults	86 (78.2)
Comprehensive eye care for infants and children	58 (52.7)
Contact lens fitting and dispensing	46 (41.8)
Cataract surgery	67 (60.9)
Refractive surgery	22 (20.0)
Retinal – vitreal surgery	15 (13.5)
Glaucoma surgery	35 (31.8)
Corneal surgery	20 (18.2)
Oculo-plastic surgery	37 (33.6)
Visual rehabilitation services	3 (2.7)
Neuro-ophthalmological services	15 (13.6)
Other	9 (8.2)
**Optometrists (N = 246)**	
Comprehensive eye care for adults	234 (95.1)
Comprehensive eye care for infants and children	200 (81.3)
Contact lens fitting and dispensing	212 (86.2)
Vision therapy	31 (12.6)
Low vision rehabilitation services	37 (15.0)
Other	20 (8.1)

Ophthalmologists’ largest patient group (42.1%) was 60–79 years of age; whereas patients 20–59 years of age were optometrists and vision rehabilitation providers largest groups (41.7% and 39.9%, respectively) (Table 4). Provider groups reported similar racial distributions for their patients, i.e., 57.1% - 61.3% white, 30.3% - 33.3% African American, and 2.3% - 6.3% Hispanic. Ophthalmologists (54.4%) and optometrists (52.6%) treated slightly more females but rehabilitation providers treated more male patients (54.6%). Ophthalmologists and rehabilitation providers reported that Medicare (50.2% and 46.9%, respectively) was the most frequent patient insurance plan; whereas for optometrists, private insurance (41.6%) was most frequent.

**Table 4:** Patient characteristics (%) by provider group

	**Ophthalmologists**	**Optometrists**	**Rehabilitation**
Age group			
< 5	6.3	4.5	2.8
5-19	9.0	19.3	16.5
20-59	26.8	41.7	39.9
60-79	42.1	25.9	25.0
80+	15.3	7.6	12.7
Race/ethnic group			
White	57.1	59.2	61.3
African American	32.6	30.3	33.3
Hispanic	5.1	6.3	2.3
Asian	2.5	3.0	0.7
Native American	0.4	0.6	0.1
Other	0.5	0.4	0.6
Gender			
Male	45.6	47.2	54.6
Female	54.4	52.6	45.4
Insurance plan			
Medicare	50.2	26.6	46.9
Medicaid	16.2	15.2	30.1
Private insurance	36.7	41.6	19.4
No insurance	4.1	18.1	23.3
Others	2.5	3.4	0
Diabetes	27.3	22.3	27.9
Adhere to guidelines	61.4	52.9	N/A
Low vision	14.4	6.6	66.7

Table 5 presents specific services offered by vision rehabilitation providers. About half (49.4%) of those responding provided in home service. The majority (63.3%) provided training in the use of assistive devices, followed by training in strategies to perform everyday visual tasks (55.7%) and orientation and mobility training (43.0%). Many patients had difficulties in reading (63.8%) and writing (48.3%), followed by difficulties in driving (55.2%) and mobility (50.1%).

**Table 5:** Services provided by vision rehabilitation providers, N (%) and patients (%) with specific difficulties or problems

**Provide in-home services**	**40 (49.4)**
**Services provided**	
Training in the use of assistive devices (e.g., optical, non-optical)	50 (63.3)
Orientation and mobility training	34 (43.0)
Eccentric viewing training or training in preferred retinal loci	15 (19.0)
Scanning strategy training	20 (25.3)
Training in strategies to perform everyday visual tasks (e.g., household activities, managing money, preparing meals)	43 (55.7)
Psychological or counseling services	10 (15.2)
Support groups (for clients and/or families)	26 (32.9)
Social work services	6 (7.6)
Driving rehabilitation	3 (3.8)
Home-based visits for education or training	32 (40.5)
Vocational rehabilitation or career counseling services	29 (36.7)
Training in the use of computers and software	31 (39.2)
Other	18 (22.2)
**Patients with specific difficulties or problems**	
Reading	63.8
Writing	48.3
Financial management	31.1
Other detail near tasks	37.3
Independent living	40.8
Mobility	50.1
Driving	55.2
Identification of objects, people, events from a distance	48.8
Self care/domestic activity	32.8
Emotional or psychological adjustment	35.4

## Discussion

The current survey presents details about the county of location of Alabama eye care providers, and among survey participants, descriptive information about demographics, and practice and patient characteristics.

Due to an aging population, future demand for eye care services is likely to outpace available resources. In Alabama this shortage will potentially be compounded by a concurrent increase in diabetes-related eye conditions, i.e., diabetic retinopathy, glaucoma, and cataracts [9,11-13]. Nearly all people with diabetes will eventually have diabetic retinopathy to some degree [16]. Diabetic retinopathy is the leading cause of blindness among working age adults in the United States. Alabama has a higher prevalence of diabetes than any other state, and African American Alabamians have a diabetes mortality rate that is 2.5 times greater than White Alabamians (20.6/100,000 people) [10,17]. In the current survey, ophthalmologists, optometrists, and rehabilitation providers estimated that 27%, 22% and 28%, respectively, of their patients had diabetes. Providers estimated the proportion of diabetic patients that adhered to eye care guidelines was 61.4% among ophthalmology patients and 53% among optometry patients. In addition, ophthalmologists and optometrists estimated that 20% and 14%, respectively, of their patients had diabetic eye conditions including diabetic retinopathy. Early detection and monitoring with timely treatment, e.g., retinal laser photocoagulation, can arrest or slow disease progression. Diabetic retinopathy is detected by eye care providers through a comprehensive eye examination that includes pupil dilation and examination of the fundus; however, only about half of all people with diabetes receive recommended annual comprehensive eye examinations [18].

The current survey found that many Alabama communities are geographically isolated from eye care services. People who live in rural areas have increased barriers to receive basic and specialized eye care (e.g., glaucoma and diabetic retinopathy screening, pediatric screening and comprehensive eye exams, driving rehabilitation for those with low vision), and vision rehabilitation services; when asymptomatic, they are more likely to delay eye care until their symptoms become apparent and severe [19].

The majority of participants identified themselves as white of non-Hispanic origin and few minorities were represented in any of the provider groups. Nonetheless, ophthalmologists and optometrists reported that on average 32.6% and 30.3% of the patients they treated, respectively, were African American. Vision impairment and eye disease among African Americans are two times higher than those of whites, especially uncorrected refractive error, cataract, glaucoma, and diabetic retinopathy [6-8]. Glaucoma is at least four to five times higher in African Americans as compared to persons of European descent [8,9], progressing more rapidly and appears approximately 10 years earlier in African Americans [6,20-25]. Older African Americans are less likely to receive routine, comprehensive eye care, when newly emerging eye conditions could be detected and treated in a timely fashion [26-28], which could contribute to their higher rates of eye disease and vision impairment. When they eventually enter treatment, their eye conditions are often in more advanced forms accompanied by irreversible vision impairment, and thus more difficult to treat, as compared to whites.

Research suggests that provider-patient communication and the use of preventive services can be facilitated when there is racial/ethnic concordance between providers and patients [29]. Communication problems with eye care providers have been identified by African Americans as a barrier to seeking eye care [30,31]. Research also indicates that African American physicians are more likely to care for patients in predominantly African American communities, underinsured patients, underserved patients, and those covered by Medicaid [32,33]. Thus, it is possible that an increase in the number of African American ophthalmologists and optometrists in Alabama would have positive benefits on eye health in the state.

Additional disparities are related to the decreasing numbers of general and specialty ophthalmologists. A recent analysis concluded that due to changing patient demographics, retirement, and a fixed number of ophthalmology residency slots nationwide, ophthalmology will face substantial challenges in manpower by year 2020 [34]. In addition, for optometrists there are currently no programs that provide encouragement (e.g., financial incentives, tuition coverage) to practice in rural areas. Potentially, new federal legislation, i.e., H.R. 920 (National Health Service Corps Improvement Act of 2013) to include optometrists in the National Health Service Corps student loan program that supports new graduates to work in underserved communities in exchange for educational loan repayment will increase rural optometrists [35]. Furthermore, the University of Alabama’s College of Community Health Sciences’ Rural Health Leaders Pipeline program has been successful in increasing the numbers of rural students who prepare for health and medical careers, though there is no specific focus identified on vision care [36].

By utilizing current technologies, telemedicine has the potential to fill some of the gaps in rural eye care services by removing distance barriers and providing patients remote access to eye care specialists who screen, diagnose, and manage eye diseases. Telemedicine is well suited for vision and eye disease screening services and also monitoring of disease through imaging and other specialized tests because of the low invasiveness of testing, wide spread availability and affordability of imaging technologies, high levels of diagnostic reliability [37], and ease of training of testing personnel [38]. Telemedicine has the potential to be used to screen for and monitor diabetic retinopathy, retinopathy of prematurity, age-related macular degeneration, and glaucoma [39]. Research has established the effectiveness of using digital fundus imaging with remote image interpretation for screening of diabetic retinopathy in developing nations [40], among a prison population with type 2 diabetes [41], and by the Indian Health Service for screening of Alaskan Natives [42]. Acceptance of telemedicine has increased steadily over the years stemming from its proven efficacy and cost-effectiveness, specifically in the areas of screening for diabetic eye conditions through fundus photography. Although rural areas of Alabama continue to lag behind urban areas for broadband access [43], the US Federal Communications Commission continues its support for expanded access for rural health care providers [44].

In addition to increased access, telemedicine has been shown to be efficient and effective. Relative to other screening programs, telemedicine programs may require high startup costs for infrastructure that may be supported by federal [45], and state initiatives [46]; however, successful programs that are accepted by communities, ultimately lead to decreased costs [47,48]. Thus, it is also critical to evaluate the cost-effectiveness of these programs, as compared to systems that do not rely on telemedicine.

The study was strengthened by involving a number of organizations and individuals who assisted in comprehensively identifying eye care providers currently practicing in the state of Alabama. By survey participation standards, participation was adequate among ophthalmologists (> 50%) but was less than optimal for optometrists (38.6%) and rehabilitation providers (45.5%). It is difficult to know how varying levels of participation may have influenced results. Though high participation is thought to increase the generalizability of survey results with regards to the characteristic examined, without an apparent bias, information obtained with lower participation rates may still be generalized to the background population if the participants are representative of the background population [49]. Since we are unable to characterize non-respondents, the generalizability of our findings remains unknown; in addition, we did not attempt to verify the correctness of participants’ survey responses, thus, the accuracy of the information provided is unknown.

## Conclusions

Potentially, significant benefit to the eye health of Alabamians would result through 1) Identification of strategies to increase the number of eye care providers, including more African American providers; 2) Development and implementation of strategies in the eye care system for improved detection and follow-up management of the ocular complications of diabetes; 3) Development and implementation of strategies to improve access to eye care, satellite eye care practices, telemedicine approaches and possibly transportation systems; and, (4) Scientific evaluation of these and other public eye health interventions to improve the quality of and access to eye care in Alabama.

## Competing interests

The authors declare that they have no competing interests.

## Author’s contributions

PM drafted the manuscript, contributed to the conception, design, and acquisition of the data and carried out the analysis. GM contributed to the manuscript, conception, design, and the analysis. KS acquired the data and contributed to the manuscript. CO contributed to the manuscript, conception, design, acquisition of the data and the analysis. All authors read and approved the final manuscript.

## Pre-publication history

The pre-publication history for this paper can be accessed here:

http://www.biomedcentral.com/1471-2415/14/44/prepub

## References

[B1] The Economic Impact of Vision Problems http://www.preventblindness.net/site/DocServer/Impact_of_Vision_Problems.pdf

[B2] Vision Problems in the U.S http://www.preventblindness.net/site/DocServer/VPUS_2008_update.pdf?docID=1561

[B3] Elliott AF Dreer LE McGwin G Jr Scilley K Owsley C The personal burden of decreased vision targeted health-related quality of life in nursing home residents J Aging Health 2010 22 504 521 10.1177/0898264310361368 20231730 PMC2964928

[B4] Centers for Disease Control and Prevention (CDC) Reasons for not seeking eye care among adults aged ≥40 years with moderate-to-severe visual impairment--21 States, 2006–2009 Morb Mortal Wkly Rep 2011 60 610 613 21597453

[B5] US Census Bureau http://quickfacts.census.gov/qfd/states/01000.html

[B6] Sommer A Tielsch JM Katz J Quigley HA Gottsch JD Javitt JC Martone JF Royall RM Witt KA Ezrine S Racial differences in the cause-specific prevalence of blindness in east Baltimore N Engl J Med 1991 325 1412 1417 10.1056/NEJM199111143252004 1922252

[B7] West SK Munoz B Schein OD Duncan DD Rubin GS Racial differences in lens opacities: The Salisbury eye evaluation (SEE) project Am J Epidemiol 1998 148 1033 1039 10.1093/oxfordjournals.aje.a009579 9850124

[B8] Tielsch JM Sommer A Katz J Royall RM Quigley HA Javitt JC Racial variations in the prevalence of primary open-angle glaucoma JAMA 1991 266 369 374 10.1001/jama.1991.03470030069026 2056646

[B9] Javitt JC Bean AM Nicolson GA Babish JD Warren JL Krakauer H Undertreatment of glaucoma among black Americans N Engl J Med 1991 325 1418 1422 10.1056/NEJM199111143252005 1922253

[B10] Behavioral Risk Factor Surveillance System http://apps.nccd.cdc.gov/brfss/list.asp?cat=DB&yr=2010&qkey=1363&state=All

[B11] Pasquale LR Kang JH Manson JE Willett WC Rosner BA Hankinson SE Prospective study of type 2 diabetes mellitus and risk of primary open-angle glaucoma in women Ophthalmology 2006 113 1081 1086 10.1016/j.ophtha.2006.01.066 16757028

[B12] Hiller R Sperduto RD Ederer F Epidemiologic associations with nuclear, cortical, and posterior subcapsular cataracts Am J Epidemiol 1986 124 916 925 3776974 10.1093/oxfordjournals.aje.a114481

[B13] Klein BE Klein R Wang Q Moss SE Older-onset diabetes and lens opacities. The Beaver Dam Eye Study Ophthalmic Epidemiol 1995 2 49 55 10.3109/09286589509071451 7585233

[B14] Centers of Disease Control and Prevention (CDC) Eye-Care Utilization Among Women Aged ≥40 Years with Eye Diseases ---19 States, 2006—2008 MMWR Weekly 2010 59 588 591 20489682

[B15] United Health Foundation America’s Health Ranking http://www.americashealthrankings.org/al/healthinsurance/2011

[B16] Aiello LP Gardner TW King GL Blankenship G Cavallerano JD Ferris FL 3rd Klein R Diabetic retinopathy Diabetes Care 1998 21 143 156 9538986 10.2337/diacare.21.1.143

[B17] Alabama Department of Public Health (ADPH) Diabetes in Alabama https://adph.org/diabetes/assets/DiabetesinALReport09.pdf

[B18] Lee SJ Sicari C Harper CA Livingston PM McCarty CA Taylor HR Keeffe JE Examination compliance and screening for diabetic retinopathy: a 2-year follow-up study Clin Experiment Ophthalmol 2000 28 149 152 10.1046/j.1442-9071.2000.00302.x 10981784

[B19] Odom JV Vision, visual needs, and quality of life of older people in rural environments: a report and synthesis of a meeting J Rural Health 2001 17 360 363 10.1111/j.1748-0361.2001.tb00289.x 12071562

[B20] Wilson R Richardson TM Hertzmark E Grant WM Race as a risk factor for progressive glaucomatous damage Ann Ophthalmol 1985 17 653 659 4073724

[B21] Grant WM Burke JFJ Why do some people go blind from glaucoma? Ophthalmology 1982 89 991 998 10.1016/S0161-6420(82)34675-8 7177577

[B22] Coulehan JL Helzlsouer KJ Rogers KD Brown SI Racial differences in intraocular tension and glaucoma surgery Am J Epidemiol 1980 111 759 768 7387780 10.1093/oxfordjournals.aje.a112954

[B23] Martin MJ Sommer A Gold EB Diamond EL Race and primary open-angle glaucoma Am J Ophthalmol 1985 99 383 387 3985075 10.1016/0002-9394(85)90001-7

[B24] David R Livingston D Luntz MH Ocular hypertension: A comparative follow-up of black and white patients Br J Ophthalmol 1978 62 676 678 10.1136/bjo.62.10.676 708667 PMC1043324

[B25] Wilensky JT Gandhi N Pan T Racial influences in open-angle glaucoma Ann Ophthalmol 1978 10 1398 1402 718042

[B26] Wang F Javitt JC Eye care for elderly Americans with diabetes mellitus: Failure to meet current guidelines Ophthalmology 1996 103 1744 1750 10.1016/S0161-6420(96)30432-6 8942865

[B27] Orr P Barron Y Schein OD Rubin GS West SK Eye care utilization by older Americans: The SEE project Ophthalmology 1999 106 904 909 10.1016/S0161-6420(99)00508-4 10328388

[B28] Bazargan M Baker RS Bazargan S Correlates of recency of eye examination among elderly African-Americans Ophthalmic Epidemiol 1998 5 91 100 10.1076/opep.5.2.91.1577 9672909

[B29] Saha S Komaromy M Koepsell TD Bindman AB Patient-physician racial concordance and the perceived quality and use of health care Arch Intern Med 1999 159 997 1004 10.1001/archinte.159.9.997 10326942

[B30] Owsley C McGwin G Scilley K Girkin CA Phillips JM Searcey K Perceived barriers to care and attitudes about vision and eye care: focus groups with older African Americans and eye care providers Invest Ophthalmol Vis Sci 2006 47 2797 2802 10.1167/iovs.06-0107 16799016

[B31] Ellish NH Royak-Schaler R Passmore SR Higginbotham EJ Knowledge, attitudes and beliefs about dilated eye examinations among African-Americans Invest Ophthalmol Vis Sci 2007 48 1989 1994 10.1167/iovs.06-0934 17460251 PMC1978096

[B32] Komaromy M Grumbach K Drake M Vranizan K Lurie N Keane D Bindman AB The role of black and Hispanic physicians in providing health for underserved populations N Engl J Med 1996 334 1305 1310 10.1056/NEJM199605163342006 8609949

[B33] Xu G Fields SK Laine C Veloski JJ Barzansky B Martini CJ The relationship between the race/ethnicity of generalist physicians and their care for underserved populations Am J Public Health 1997 87 817 822 10.2105/AJPH.87.5.817 9184512 PMC1381056

[B34] Lee PP Hoskins HD Jr Parke DW 3rd Access to care: eye care provider workforce considerations in 2020 Arch Ophthalmol 2007 125 406 410 10.1001/archopht.125.3.406 17353416

[B35] H.R. 920 National Health Service Corps Improvement Act of 2013 http://www.govtrack.us/congress/bills/113/hr920

[B36] The University of Alabama College of Community Health Services Rural Scholars Programs Help Meet Need for More Rural Doctors http://cchs.ua.edu/crm/rural-health-programs/rhs/

[B37] Whited JD Accuracy and reliability of teleophthalmology for diagnosing diabetic retinopathy and macular edema: a review of the literature Diabetes Technol Ther 2006 8 102 111 10.1089/dia.2006.8.102 16472057

[B38] Cuadros J Bresnick G EyePACS: an adaptable telemedicine system for diabetic retinopathy screening J Diabetes Sci Technol 2009 3 509 516 10.1177/193229680900300315 20144289 PMC2769884

[B39] Au A Gupta O The economics of telemedicine for vitreoretinal diseases Curr Opin Ophthalmol 2011 22 194 198 10.1097/ICU.0b013e3283459508 21460727

[B40] Bai VT Murali V Kim R Srivatsa SK Teleophthalmology-based rural eye care in India Telemed J E Health 2007 13 313 321 10.1089/tmj.2006.0048 17603834

[B41] Aoki N Dunn K Fukui T Beck JR Schull WJ Li HK Cost-effectiveness analysis of telemedicine to evaluate diabetic retinopathy in a prison population Diabetes Care 2004 27 1095 1101 10.2337/diacare.27.5.1095 15111527

[B42] Indian Health Services, Division of Diabetes Treatment and Prevention Fact Sheets - The IHS–Joslin Vision Network Teleophthalmology Program http://www.ihs.gov/MedicalPrograms/Diabetes/index.cfm?module=resourcesFactSheets_JVN

[B43] Severson K Digital Age is Slow to Arrive in Rural America http://www.nytimes.com/2011/02/18/us/18broadband.html?pagewanted=all&_r=0

[B44] Federal Communications Commission Rural health care support mechanism. Final rule Fed Regist 2013 78 13935 13993 23476995

[B45] US Department of Health and Human Services Health Resources and Services Administration http://www.hrsa.gov/ruralhealth/about/telehealth/

[B46] California Telehealth Network California Telehealth Network http://www.caltelehealth.org/press-release/uc-davis-co-manage-22-million-fcc-telehealth-grant

[B47] Whited JD Datta SK Aiello LM Aiello LP Cavallerano JD Conlin PR Horton MB Vigersky RA Poropatich RK Challa P Darkins AW Bursell SE A modeled economic analysis of a digital tele-ophthalmology system as used by three federal health care agencies for detecting proliferative diabetic retinopathy Telemed J E Health 2005 11 641 651 10.1089/tmj.2005.11.641 16430383

[B48] Maberley D Walker H Koushik A Cruess A Screening for diabetic retinopathy in James Bay, Ontario: a cost-effectiveness analysis CMAJ 2003 168 160 164 12538543 PMC140424

[B49] Johnson TP Wislar JS Response rates and nonresponse errors in surveys JAMA 2012 307 1805 1806 10.1001/jama.2012.3532 22550194

